# Understanding the Role of the Transcription Factor Sp1 in Ovarian Cancer: from Theory to Practice

**DOI:** 10.3390/ijms21031153

**Published:** 2020-02-09

**Authors:** Balachandar Vellingiri, Mahalaxmi Iyer, Mohana Devi Subramaniam, Kaavya Jayaramayya, Zothan Siama, Bupesh Giridharan, Arul Narayanasamy, Ahmed Abdal Dayem, Ssang-Goo Cho

**Affiliations:** 1 Human Molecular Cytogenetics and Stem Cell Laboratory, Department of Human Genetics and Molecular Biology, Bharathiar University, Coimbatore 641046, India; 2 Department of Zoology, Avinashilingam Institute for Home Science and Higher Education for Women, Coimbatore 641043, India; geneticsmaha@gmail.com (M.I.); kaavyajayaramayya@gmail.com (K.J.); 3 Department of Genetics and Molecular Biology, Vision Research Foundation, Sankara Nethralaya, Chennai 600006, India; geneticmohana@gmail.com; 4 Department of Zoology, School of Life-science, Mizoram University, Aizawl 796004, Mizoram, India; zothans@gmail.com; 5 R&D Wing, Sree Balaji Medical College and Hospital (SBMCH), BIHER, Chromepet, Chennai 600044, Tamil Nadu, India; bupeshgiri55@gmail.com; 6 Disease Proteomics Laboratory, Department of Zoology, Bharathiar University, Coimbatore 641046, Tamil Nadu, India; swamyarul@gmail.com; 7 Molecular & Cellular Reprogramming Center, Department of Stem Cell & Regenerative Biotechnology, Konkuk University, Seoul 05029, Korea; ahmed_morsy86@yahoo.com

**Keywords:** ovarian cancer, therapeutics approach, cellular reprogramming, transcription factor, Sp1

## Abstract

Ovarian cancer (OC) is one of the deadliest cancers among women contributing to high risk of mortality, mainly owing to delayed detection. There is no specific biomarker for its detection in early stages. However, recent findings show that over-expression of specificity protein 1 (Sp1) is involved in many OC cases. The ubiquitous transcription of Sp1 apparently mediates the maintenance of normal and cancerous biological processes such as cell growth, differentiation, angiogenesis, apoptosis, cellular reprogramming and tumorigenesis. Sp1 exerts its effects on cellular genes containing putative GC–rich Sp1–binding site in their promoters. A better understanding of the mechanisms underlying Sp1 transcription factor (TF) regulation and functions in OC tumorigenesis could help identify novel prognostic markers, to target cancer stem cells (CSCs) by following cellular reprogramming and enable the development of novel therapies for future generations. In this review, we address the structure, function, and biology of Sp1 in normal and cancer cells, underpinning the involvement of Sp1 in OC tumorigenesis. In addition, we have highlighted the influence of Sp1 TF in cellular reprogramming of iPSCs and how it plays a role in controlling CSCs. This review highlights the drugs targeting Sp1 and their action on cancer cells. In conclusion, we predict that research in this direction will be highly beneficial for OC treatment, and chemotherapeutic drugs targeting Sp1 will emerge as a promising therapy for OC.

## 1. Introduction

Ovarian cancer (OC) has been identified as the deadliest multidrug resistant cancer among females, especially at their perimenopausal stage [1]. Reports suggest that OC is the second most common reproductive cancer among women in India [2]. Although OC is a single disease, its clinical pathway is mostly intercalated by other tumor types having different prognosis stages, morphologies, and molecular and epigenetic backgrounds [3]. Presently, there are no early-stage treatment options for OC since the early symptoms cannot be comprehended. Due to high prevalence and the continuously rising incidence, OC poses a major threat to personal health as well as the health care system [1]. Despite ongoing efforts to detect OC, specific diagnostic biomarkers are yet to be identified [4]. It is evident that transcription factors (TFs) play a pivotal role in the regulation of cellular functions, including cell activation, repression, and alteration of gene expression. Any dysfunctional activation or inactivation of TFs may result in cellular induction of tumorigenesis [4]. Mutations in cancer is caused due to changes in various proteins functions and transcriptions factors which are controlling the protein, thus changing the phenotypes in human [5]. Bookmarking of mitosis constitutes a mechanism that transmits transcriptional patterns by cell division. Bookmarking factors, comprising a subset of TFs, and multiple histone modifications retained in mitotic chromatin facilitate reactivation of transcription in the early G1 phase [6]. It is possible that Sp1 phosphorylation may change its interaction with other transcription factors [7]. Specificity protein 1 (Sp1) is one such ubiquitous and multifunctional TF from the Sp/Kruppel-like family (KLF) TFs, which are the major forms of zinc-finger DNA binding proteins (also known as specificity protein 1 and TSFP1) belonging to a member of the KLF TFs [8]. The Sp1 gene was first cloned by Kadonaga and co-workers, and the various functional domains of Sp1 were determined in a series of in vitro and whole cell assays. It was first identified by cell fractionation procedures and shown to interact with GC and GT oligonucleotide sequences that are typically found in diverse viral and cellular gene promoters [9]. Sp1 was the first constitutive eukaryotic transactivator of both housekeeping and TATA genes and it has been observed to be high in epithelial ovarian cancer [10]. Dynan and Tjian initially observed that Sp1 can selectively transactivate the early and late simian virus 40 promoters without influencing many other promoters and regulate the expression of thousands of genes involved in the control of a variety of cellular processes, such as cell growth, differentiation, apoptosis, angiogenesis, and immune response. Furthermore, the authors observed that the promoter of Sp1 activated the SV40 and increased transcription by 40-fold, while inhibition of adenovirus delayed the promoter binding by 40% [11]. Similar studies identified that Sp1 binds to the *dhfr* promoter resulting in gene expression for de novo synthesis of purines, thymidylate and glycine and its role as promoter for SV40 [12].

It is important to understand how a complex factor such as Sp1 is involved in basal transcriptional regulation in various genes. The encoded protein is involved in many cellular processes, including cell differentiation, cell growth, apoptosis, immune responses, response to DNA damage, and chromatin remodeling. Post-translational modifications such as phosphorylation, acetylation, glycosylation, and proteolytic processing significantly affect the activity of this protein, which can be an activator or a repressor [13]. When SP1 is overexpressed and contributes to the malignant phenotype of a variety of human cancers by upregulating genes that enhance proliferation, invasion, and metastasis as well as stem-ness and chemoresistance [14]. Investigations have revealed that both upregulation and downregulation of Sp1 can modulate several oncogenes, thus regulating the metastasis and tumor growth in OC [15,16]. It is also found that Sp1 supports angiogenesis and opposes apoptosis in cancer cells, thereby aggravating tumorigenesis. In a recent study, it was found that high Sp1 expression leads to autophagic flux and increased tumorigenesis [17]. These lines of evidence support that a more in-depth knowledge about Sp1 would increase the options for treating OC. This review synopsizes the fundamental role of Sp1 in normal cells and its precise role as a regulator for OC tumorigenesis. In conclusion, we suggest Sp1 as one of the best potent drug targets to treat OC.

## 2. Genetic Makeup and Structure of Sp1

**Figure 1:**
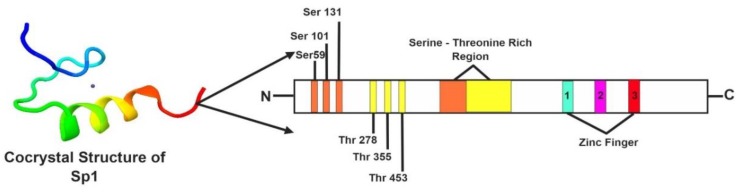
The Co-crystallized Structure of specificity protein 1 (Sp1). The Sp1 protein is 785 amino acids long with a molecular weight of 81 kDa. The figure depicts the co-crystallized structure of Sp1, where the protein has three highly homologous C2H2 –type zinc finger motif-rich regions. This region is responsible for the binding to GC-rich DNA motifs (such as 5′-G/T-GGGCGG-G/A-G/A-C/T-3′ or 5′-G/T-G/A-GGCG-G/T-G/A-G/A-C/T-3′) and for the regulation of gene transcription of a large number of genes involved in various processes such as response to DNA damage, chromatin remodeling, cell growth, apoptosis, differentiation, and immune responses. The transcriptional activity of Sp1 can be modulated by several post-translational modifications including phosphorylation, acetylation, ubiquitylation, sumoylation, and glycosylation. The phosphorylation sites such as Ser59, Ser101, Ser131, Thr278, Thr335, and Thr453, were indicated in the figure. (Figures have been created with BioRender.io https://biorender.com/).

The gene encoding Sp1 is located in the q arm of the 12th chromosome in humans [8]. According to the AceView database, the sequence of the *Sp1* gene is supported by 512 sequences from 447 cDNA clones [18]. In humans, the Sp1 gene produces four transcripts generated by alternative splicing with a transcript length of 602 bps and a translational length of 162 residues. The Sp1 protein is almost 785 amino acids long with a molecular weight of 81 kDa. The earlier structured domain region of Sp1 was analyzed using standard homo-nuclear two-dimensional nuclear magnetic resonance imaging techniques, revealing a classical Cys2-His2 type fold in the Sp1 domain [19,20]. Sp1 contains three highly homologous C2H2 regions, which exhibit direct binding to DNA, thus enhancing gene transcription [21]. Sp1 has four unstructured domains A, B, C, and D. The defining feature of Sp1-like/KLF proteins is a highly conserved DNA-binding domain (more than 65% sequence identity among family members) at the carboxyl terminus that has three tandem Cys2His2 zinc-finger motifs [22]. The DNA-binding domain (C-terminal domain) of the Sp1-like transcription factor family is highly conserved, whereas the N-terminal regions of the proteins are more divergent. Interestingly, it is through this domain that many of these transcription factors regulate transcription [23]. The two main transactivating domains (TAD) of Sp1 are A and B, which are capable of direct interaction with the components of transcription machinery such as TATA-binding protein (TBP) and TBP-associated factor 4 (TAF4) [24]. The C domain is not indispensable, but it is highly charged and supports DNA binding and transactivation. The D domain, also known as the C-terminal region of SP1 has multimeric domains and is responsible for the binding of consensus sequences such as 50-(G/T) GGGCGG(G/A)(G/A)(G/T)-30 [25]. The N-terminal region is a small inhibitory domain (IB), which mainly regulates functions of domains A and B, and is linked with a serine-threonine-rich region [24]. The co-crystallized structure of Sp1 has been depicted in Figure 1.

## 3. Regulation of Sp1

The uniqueness of the Sp1 TF is that it not only initiates transcription but also regulates the activation or repression processes. Growing evidence suggests that the transcriptional activity and stability of Sp1 is influenced by its post-translational modifications (PTMs). Sp1 undergoes acetylation, sumoylation, ubiquitylation, and glycosylation after translational [26,27]. Acetylation of Sp1 takes place in the DNA binding domain [28]. Glycosylation occurs at the at O-GlcNAc linkages at the Ser and Thr residues in Sp1, which can either induce or suppress DNA binding and transcription [29]. Sumoylation, occurring in the Lys16 region, controls the transcription of Sp1 by instigating alterations in the chromatin structure, making the DNA inaccessible for transcription [30]. The proteasomal degradation of Sp1 is carried out by the ubiquitylation, where Sp1 is directly bonded with the binding motif of the beta-TCRP ubiquitin ligase complex [31]. Thus, the impact and influence of PTMs on the transcriptional activity of Sp1, and, in particular, the modulation of its affinity for DNA/proteins, have helped clarify the mechanisms related to tumorigenesis.

## 4. Role of Sp1 in the Normal Cell Cycle and OC Tumorigenesis 

**Figure 2:**
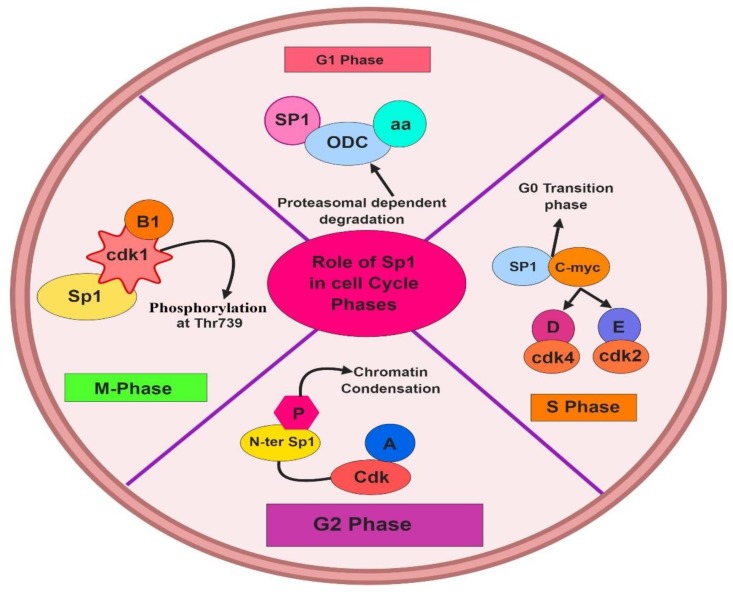
Role of Sp1 in the cell cycle. Improper functioning of the cell cycle and its checkpoints are generally a key factor for cancer cell growth. Some major interactions between the activity of transcription factor Sp1 and different components of the cell cycle phases, and its coordinating regulators have been depicted in this figure. In the G1 phase of the cell cycle, an elevated level of nuclear Sp1 augments the proliferation of Sp1-responsive genes such as ODC and cyclin D1. In M-phase of cell cycle, Sp1 TF acts as a mitotic substrate of CDK1/cyclin B1. In G2 phase of cell cycle Sp1 undergoes phosphorylation due to cyclin A-CDK complexes. In the transition periods from G1/S phase, Sp1 stimulates cyclin D/Cdk4, cyclin E/Cdk2, E2f–1, and c–myc genes. These interfaces often result into an abnormal cell cycle progression and possibly into cancer cell growth and progression. (Figures have been created with BioRender.io https://biorender.com/).

Sp1 has a key role in regulating cyclins, CDKs, and CDKIs, which are critical components of cell cycle machinery [32]. In the G1 phase of the cell cycle, the proteasome dependent degradation mechanism is correlated directly with elevated levels of nuclear Sp1, which also augment the proliferation of Sp1-responsive genes such as ODC and cyclin D1 [33]. Sp1 is a mitotic substrate of CDK1/cyclin B1, which is phosphorylated at Thr739 of CDK1/cyclin B1 in the M-phase of the cell cycle [26]. In vitro and in vivo studies reveal that the N-terminal region of the Sp1 protein undergoes phosphorylation due to the formation of cyclin A–CDK complexes in the G2 phase of the cell cycle, reducing DNA binding and facilitating chromatin condensation [34,35]. During the transition period of the G1/S phase, Sp1 induces cyclin D/Cdk4, cyclin E/Cdk2, E2f–1, and c–myc genes [36]. The roles played by Sp1 in cell cycle phases have been depicted in Figure 2. Thus, Sp1 has a putative job in cell cycle regulation which may result into tumor development and progression upon disruption. 

**Figure 3:**
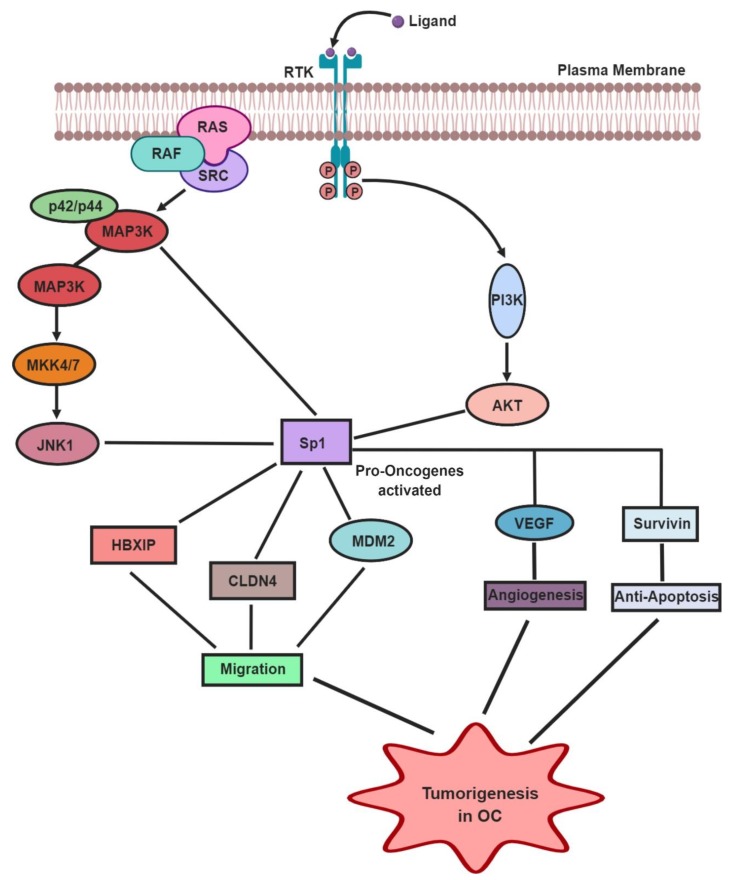
Sp1 mediated tumorigenesis in ovarian cancer (OC). Evasion in the Sp1 transcriptional activities at various levels has been found as a cause for tumorigenesis in OC cells. In OC cells, the transcription activity of Sp1 can be enhanced by various oncogenes like Ras, Src, and Raf especially through MAP3K, PI3K/AKT, and JNK1 pathway. In addition, Sp1 affects the tumorigenesis by activating the pro–oncogenes such as HBXIP, CLDN4, and MDM2, resulting in the migration of OC cells. Furthermore, Sp1 is seen to up-regulate VEGF and survivin genes, leading to angiogenesis and anti–apoptosis in the OC cells (Figures have been created with BioRender.io https://biorender.com/).

Tumorigenesis can be defined as an uncontrolled cell cycle progression. Defects in the Sp1 transcriptional activities act as a cause for tumorigenesis in many types of cancers, such as ovarian, breast, and gastric cancer. The transcription activity of Sp1 in cancer cells is enhanced by various oncogenes like Ras, Src, and Raf, especially through the p42/p44 mitogen-activated protein kinase/extracellular signal-regulated kinase (MAPK/ERK) pathway [37]. Sp1 expression levels are associated with poor disease prognosis, especially in OC [38]. Many studies note that Sp1 predominantly regulates oncogenes such as XIP, Claudin 4 (CLDN4), cyclin E, KLF8, and vascular endothelial growth factor (VEGF), which contribute to OC tumorigenesis [39,40,41,42,43]. Xu et al. observed that hepatitis B X–interacting protein (HBXIP), a novel oncoprotein, when bound with Sp1 TF, was highly expressed in OC cells [37]. Furthermore, it was also found that S-phase kinase-associated protein 2 (Skp2), another oncoprotein, promotes the migration of OC cells via Sp1 TF [37]. High levels of another OC oncogene, CLDN4 are generally controlled by epigenetic alterations in the promoter region of Sp1 [39]. A case-control study in Caucasians revealed that the MDM2 proto-oncogene showed a T309G polymorphism, enhancing its binding affinity to Sp1, thereby elevating the chances of OC tumorigenesis (Figure 3) [44]. The activation of various tumor signaling pathways exposes the cells to stressful environmental conditions such as oxygen and nutrients deprivation. Sp1 and hypoxia–inducible factors (HIF2), a ubiquitously expressed TF in ovarian clear cell carcinoma (CCC), it was observed that activation of long chain fatty acid (LCFA) resulted into starvation and hypoxia type of micro-environment in ovarian CCC cases [45]. Thus, it is suggested that knocking down or inhibiting the levels of Sp1 in OC cells can decrease tumor formation, tumor growth, and metastasis.

In OC, the most frequently altered pathways include JNK1 (c–Jun N–terminal kinase 1) pathways, MAPK signaling, and the PI3K/AKT pathway (Figure 3). Rhox5 homeobox protein, which is highly expressed in the granulosa cells of ovaries, is upregulated by TFs such as ETS and Sp1 via various pathways such as JNK, MAPK8, and RAS [46]. The metabolism in cancer cells is mainly controlled by the PI3K/AKT pathway by Sp1-mediated transactivation of various oncogenes [47]. Milanini-Mongiat et al. [48] found that JNK1 and JNK2 pathways control the activation as well as upregulation of Sp1 [48], eventually leading to the activation of oncogenes and tumorigenesis. Further, it was also shown that the p42/p44 MAPK pathway alone can phosphorylate Sp1 at the T453-739 region, enhancing the Sp1–DNA (promoter) interactions, ultimately resulting in OC progression [48]. On similar grounds, it was also reported that CD147, an important biomarker found in OC, stimulates Sp1 phosphorylation at T453 and T739 sites, especially through the PI3K/AKT and MAPK/ERK signaling pathways [49]. Another interesting fact about the p42/p44 MAPK pathway is that it can activate stress factors such as hypoxia and release Reactive Oxygen Species (ROS) and Nitric Oxide (NO) [50], which triggers Sp1 into activating various oncogenes [51]. Thus, the given literature suggests that major cancer-associated signaling pathways trigger Sp1 to activate various oncogenes and support the development and progression of OC.

## 5. Effect of Sp1 TF in Angiogenesis and Anti-Apoptosis in OC

Angiogenesis is the process of formation of new blood vessels within the cells, and it is an important prognostic factor for the pathophysiological conditions seen in OC cells. VEGF is one of the genes normally linked with most of the angiogenic processes in cancer cells. A few studies have proven that Sp1 promotes angiogenesis in OC via induction of VEGF expression by directly binding to its promoter site [52,53,54]. It has also been suggested that Sp1 upregulates VEGF via the AKT pathway, eventually initiating angiogenesis for the invasion of tumor cells [55,56]. Sue et al. found that the upregulation of Sp1 in the SKOV3 cell line enhances the expression of VEGF, and initiates angiogenesis, thus provoking the malignancy of OC [57] (Figure 3).

Another significant factor for OC tumorigenesis is the dysregulation of apoptosis, as it can activate the invasion, prognosis, and resistance to chemotherapy in OC cells. Apoptosis is a type of programmed cell death, where the pro- and anti-apoptotic proteins control the life and the death switch of the cell. The *survivin* gene belongs to the inhibitor of apoptosis protein (IAP) family, which is a key agent for the anti-apoptosis process. The promoter region of the surviving gene has GC–rich sites, which are known to be the binding site for Sp1 (Figure 3) [58]. Overexpression of the Sp1 TF has been shown to lessen the level of apoptosis in cancer cells [59]. Interestingly, it has been observed that the downregulation of Sp1 induced by tolfenamic acid (TA) can promote the apoptosis in OC cells [60]. These observations suggest that Sp1 has a major role in promoting angiogenesis and anti-apoptosis in OC cells. Further research is necessary to understand the exact mechanism underlying OC.

## 6. Sp1 as a Therapeutic Target in OC

Ovarian cancer (OC) has a very poor prognosis because of delayed diagnosis in most of the patients and resistance to some cytotoxic drugs. A major obstacle that jeopardizes OC chemotherapeutic treatment is multidrug resistance (MDR) [1]. A remarkable number of studies have revealed the importance of Sp1 in this regard, as it regulates potent drug targets as well as promoter genes that are overexpressed in OC [40,61]. Targeting Sp1 TF directly with the help of Mir-128 and Mir-377 reduces the rate of cell cycle, proliferation, and invasion of the cancer cells [62]. Thus, it is evident that Sp1 can be exploited as a suitable drug target to treat OC [63]. Until now, very few drug compounds or natural extracts have been used to specifically target Sp1 for treating various cancer forms. The drugs used so far are enlisted in Table 1 [64,65,66,67,68,69,70,71,72,73,74,75,76,77,78,79,80,81,82,83]. One of the popularly used compounds for treating OC is mithramycin A (MTA), an aureolic acid antibiotic that is a natural polycyclic aromatic polyketide made from diverse species of *Streptomyces* [84]. The interaction of MTA with the GC–rich regions of the promoter results in the blocking of Sp1 binding sites in cancer cells [64,85]. Besides, MTA and its analogs can downregulate most of the Sp1-regulated genes in OC cell lines [86]. In a functional study, it was found that two new analogs of MTA, namely MTMSDK and MTM–SK, hindered the growth of OC cells in xenografts via inhibition of Sp1–based transcription [87]. Another efficient analog of MTA is demycarosyl-3D-ß-D-digitoxosyl-mithramycin SK (DIG–MSK), as it can inhibit Sp1-mediated transcription, mRNA expression, and various other genes regulated by Sp1 that have a pivotal role in OC, like VEGFA, BCL2L1 (Bcl-2-like 1; Bcl-XL), human telomerase Reverse Transcriptase (hTERT), BRCA2, and MYC [88]. Similar to this study, Vizcaino et al. also observed that DIG–MSK can downregulate the binding of Sp1 to pro-oncogenes in OC cells [89]. Another commonly used drug for treating OC is tolfenamic acid (TA) a non-steroidal anti-inflammatory drugs (NSAID), which generally induces the degradation of Sp protein. An earlier study noted that TA has positive effects on OC tumor growth in mice, including degradation of the Sp1 protein, leading to a decrease in cell proliferation, while encouraging apoptosis and cell cycle arrest [60]. Betulinic acid (BA) is an anti-cancer drug that can inhibit topoisomerase and has also been used for downregulation of Sp1 expression and its regulated pro-oncogenes in various cancer cells [89,90]. Currently, the trending remedial route to treat any form of cancer is by micro RNA-mediated targeting. Interestingly, it has been observed that the introduction of miR–14 into OC cell lines downregulates Cdk6, Sp1, and P-glycoprotein (P-gp), resulting in a more efficient penetration of drugs like paclitaxel into the targeted OC cells [91]. In a recent study, the authors have confirmed that the direct target of miRNA while targeting OC cells is the KLF12 which is an antagonist for Sp1 [92]. Similarly, in another recent study, the authors have found a new signaling pathway named as miR-141/KLF12/Sp1/survivin which enables to improve anoikis resistance and acts as potent therapy for OC patients [93].

Drugs designed by applying the mechanism-based criteria, such as targeting important pro-oncogenes, their associated pathways, and their initiators, are approved for clinical use fairly easily. Various potential strategies are available for blocking or inhibition of Sp1. These include activation of ROS, proteasome, caspases, the cannabinoid receptors, and targeting Sp1 binding GC–rich regions. Recently, it has been reported that targeting Sp1 expression at the G2/M phase of cell cycle by radiotherapy helps reduce tumor development [94]. Interaction between the long non-coding RNA ZFAS1 and miR-150-5p suppresses the expression of Sp1 in epithelial OC cells [95]. An antipsychotic drug, penfluridol, is currently used as an anti-cancer drug since it can downregulate the TFs like Sp1, Sp3, and Sp4 [96]. It was found that penfluridol inhibits cancer cell growth by suppressing expressions of α6- and β4-integrins, primarily regulated by the Sp1 along with orphan nuclear receptor 4A1 (NR4A1) [97]. In OC cells, the interaction between NR4A1 and Sp1 forms a negative feedback loop, unbalancing the regulation of growth or survival genes such as survivin and EGFR through their proximal GC–rich promoter elements [98]. Targeting NR4A1 by an antagonist leads to the normal expression of Sp1 and redox genes for maintaining low levels of oxidative stress in the tumor microenvironment [99,100]. According to a few studies, BA inhibits the growth of cancer cells by stimulating proteasome-dependent downregulation of Sp1, Sp3, and Sp4 [89,90,101]. Most of the xenograft models used to study the effect of drugs on targeting the functions of Sp1 in treating OC are the OC cell lines such as SKOV3, ES2, OVCAR-3, A2780, and SKOV3 cells, the related paclitaxel-resistant cell lines, A2780/PTX and SKOV3/PTX, a human breast cancer cell line (MCF-7), the adriamycin-resistant cell line (MCF-7/ADM), and normal human ovarian epithelial cells (HOEC). Thus, the discovery of such DNA–binding compounds and various drug-targeting mechanisms, which specifically target Sp1 TF, could pave ways for better treatment options in OC cases.

## 7. Influence of Sp1 on Cellular Reprogramming

**Figure 4:**
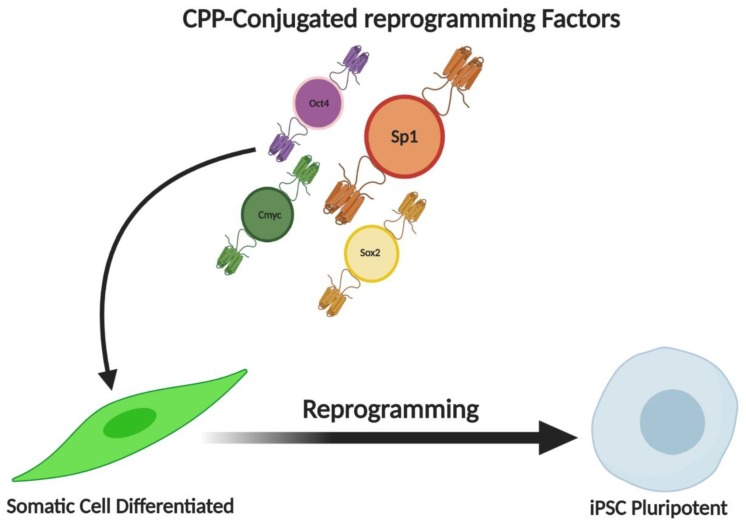
Potential role of Sp1 transcription factor in cellular reprogramming. Sp1 is one of the most important transcription factors that are associated with the major reprogramming factors Sox2, c-Myc, and Oct4, that are used for iPSC induction. Sp1 TF is an analog for Klf4 TF, thus Sp1 do play a major role in reprogramming process of cancer stem cells. Sp1 was also considered as a vital component involved in the conversion of somatic cells into pluripotent cells making it a target to increase the efficiency of reprogramming.

Stem cells and Cancer stem cells appear to have similar regulatory signals in their microenvironments that contribute to their reprogramming and proliferative potential. But the mechanisms involved in reprogramming continue to remain enigmatic. Recently, switch genes have been identified, that convert glioblastomas from stem-like cells to differentiated forms. Sp1 transcription factors, were recognized as central regulators of the switch genes, displaying their potential role in cellular reprogramming [102]. Sp1 is one of the most important transcription factors that are associated with the major reprogramming factors Sox2, c-Myc, and Oct4, that are used for IPSC induction (Figure 4) [103]. Similarly, when goats IPSCs were generated, it was observed that the core genes previously mentioned had Sp1 binding sites in their core promoter. Making Sp1 an accomplice in reprogramming [104]. Sp1 was also considered a vital component involved in the conversion of fibroblasts into neurons, making it a target to increase the efficiency of reprogramming protocols [105]. Similarly, MiR-590 a direct repressor of Sp1 has also been studied in conversion studies. When miR-590-mediated repression of Sp1 was done on human cardiac cells, it was found that it significantly upregulated the associated genes and promoted cardiac cellular reprogramming, showing that Sp1 may be an intermediary in this step [106] The E-Ras/JNK signalling is a critical mechanism to generate iPS cells by transduction of 4 factors. E-Ras was found to enhance binding of Sp1 on the cyclins to promote cell proliferation and reprogramming. This is identified as a way to increase the efficiency of IPSC derivation protocols [107]. Regeneration existing in the epicenter of reprogramming, has been studied for decades. It was found that Sp1 could play a potential role in limb regeneration, making it a focus worthy participant of reprogramming [108].

Cellular reprogramming is a process where any dysfunctions in the cells can be retrieved by erasing any kind of epigenetic alterations in the somatic cells using Induced Pluripotent Stem Cells (iPSCs). This process of cell reprogramming or repairing was first anticipated by Dr. John Gurdon during an experiment on the cloning process of somatic cells in the *Xenopus laevis* [109]. The effectiveness of reprogramming is almost less than 1%. The reason for this is because the major change which occurs during reprogramming process is the changes in the epigenetic status which includes DNA methylation, histone and acetylation modifications. To overcome these issues, the most promising method is to use TF induced reprogramming to iPSC because it makes the process very simpler and sturdy [110]. Recently it has been reported that iPSC technology is been evolving as a promising therapy to treat various diseases such as cancer, neurological disorders, cardiovascular diseases and so on [111]. Recently Saha et al., has pointed out that the biology of reprogramming in the framework of replicative age of the cells needs more appropriate stimulating agents such as a more reliable TF to reduce the epigenetic stress caused during reprogramming process [112]. The durability of the cancer cells was strong due to the presence of stem cell-like property in them which is known as CSCs [1]. Thus, recent functional study has mentioned that targeting the CSCs in the cancer cells using either molecular medicine or TFs-induced reprogramming of iPSCs would provide a positive effect in blocking the progression and invasion of the cancer cells [113]. During cellular reprogramming, the nucleosome is occupied with the binding of Oct4 and Sox2 in the embryonic stem cells (ESCs). Moreover, the Sp1 TF which is an analog for Klf4, interacts with the DNA in the nucleosome during cellular reprogramming [114]. It has also been suggested that, during reprogramming to iPSCs, the DNA interaction via Sp1 TF does not undergo any epigenetic alterations such as DNA methylation [115]. Thus, these studies give us a brief idea that Sp1 can work as an important TF for modulating cellular reprogramming and can be used for future OC treatment.

## 8. Future Perspective

Till now the only valid and trust-worthy tool to detect cancer is the cytogenetic assay, where the chromosomal aberrations in the peripheral lymphocytes of the patients [116,117]. Despite the thorough characterization of the regulatory mechanisms of various therapeutic actions in OC, several factors associated with the poor prognosis and survival rates have yet to be elucidated. Currently, advanced chemotherapy is commonly used to treat OC. However, it is often ineffective due to the occurrence of multi-drug resistance. Over the past few decades, the elucidation of the role of Sp1 in OC has altered the scope of cancer research. The increasing recognition and better understanding of Sp1’s pivotal role in regulating the housekeeping genes and basic biological functions suggests that it could be a novel therapeutic target in OC. The Sp1 transcription factor has been increasingly evaluated over the past few years and has emerged as an intensive unit of study in cancer cells owing to its ubiquitous nature, its major role as a basal transcriptional regulator, and as a promoter of tumor progression. In the near future, development of therapies based on specific DNA binding interactions can be designed to prevent disease progression and to step up the survival rates in OC.

## 9. Conclusions

In conclusion, we suggest that novel medicinal plant-based compounds must be developed for suppressing the oncogenic functions of Sp1 by targeting its specific sites in OC. A higher number of clinical or phase trials must be carried out to ascertain the effect of OC therapy routines on Sp1 signaling and to develop strategies for modifying the Sp1–targeted survival response. Finally, the biggest challenge is to deliver adequately dosed interventions, taking into account the many sources of interference for a specific, tissue-targeted or cell-targeted effect in OC.

## Figures and Tables

**Table 1:** Sp1-targeting Drug Compounds.

#	Compound Name	Binding Site	Reactions	References
**1**	Mithramycin A	Sp1 gene promoters	Alters Sp1 and DNA interactions	[64,65,66,67]
**2**	Daunorubicin	Binds to DNA with higher affinity	Inhibits Sp1-DNA interactions & gene transcription	[68,69,70]
**3**	WP631(bis-intercalating anthracycline)	Binds to DNA with higher affinity	Efficient inhibitor for transcription initiation of Sp1 containing binding sites & Sp1—activated transcription	[69,71]
**4**	Doxorubicin	Activates promoter of Cdc25B	Inhibits Sp1 binding & increases NF-Y binding to promoter and keeps P53 alive	[72]
**5**	Hedamycin	Down—regulates surviving expression	Abolishes Sp1 binding to putative binding elements & modulates viability of cancer cells	[73]
**6**	Elsamicin A	DNA—protein interactions in c–myc promoters	Affects Sp1 binding in a dose—dependent manner	[74]
**7**	Actinomycin D	DNA—protein interactions	Sp1 TFs induce TNF expressions on angiogenic factors in cancer cells	[75]
**8**	Tolfenamic acid	Drug—DNA interaction	Increases ubiquitination of Sp1 as well as the proteasome–dependent degradation (downregulates Sp1)	[76,77]
**9**	Aspirin	Cells that response to sequestration of zinc ions	It induces caspase–dependent cleavage of Sp1 protein factors	[78]
**10**	Arsenic trioxide	Human telomerase reverse transcriptase (hTERT) gene	Suppresses transcription of hTERT gene through regulation Sp1 TF	[79]
**11**	Curcumin (diferuloylmethane)	Not described	Induces proteasome–dependent down—regulation of Sp1 proteins	[80,81]
**12**	Betulinic acid	Not described	Decreases expression of Sp1 TF in cancer cells	[82]
**13**	Resveratrol (3,5,4′-trihydroxy-trans-stilbene)	Not described	Inhibits cell growth and Sp1 TF directly	[83]

Sp1—Specificity Protein 1; TF—Transcription Factor; WP631—Bis-intercalating Anthracycline; Cdc25—Cell Division Cycle 25B; NF-Y—Nuclear Factor Y; C–myc—Myc proto-oncogene; TNF—Tumor necrosis factor; hTERT—Human telomerase reverse transcriptase.

## Abbreviations

OC	Ovarian cancer
Sp1	Specificity protein 1
KLF	Kruppel-like family
TF(s)	Transcription factors
VEGF	Vascular endothelial growth factor
CCC	Ovarian clear cell carcinoma
JNK1	Jun N—terminal kinase 1
MAPK/ERK	mitogen-activated protein kinase//extracellular signal-regulated kinase
MDR	Multidrug resistance
IAP	Inhibitor of apoptosis protein
MTA	Mithramycin A
BCL2L1	Bcl-2-like 1
hTERT	Human telomerase Reverse Transcriptase
CLDN4	Claudin 4
HIFs	Hypoxia—Inducible Factors
LCFA	Long Chain Fatty Acid
HBXIP	Hepatitis B X—interacting protein
Skp2	S—phase kinase—associated protein 2
ROS	Reactive Oxygen Species
NO	Nitric Oxide
Raf	Rapidly Accelerated Fibrosarcoma
Sox2	SRY-Box Transcription Factor 2
Oct4	Octamer-binding transcription factor 4
Klf4	Kruppel-like factor 4
JNK1	c-Jun N-terminal kinases
HBXIP	Hepatitis B X-interacting protein
CLDN	Claudin
VEGF	Vascular endothelial growth factor
ODC	Ornithine decarboxylase
CDK	Cyclin-dependent protein kinases
